# Changes in Leaf Anatomical Traits Enhanced Photosynthetic Activity of Soybean Grown in Hydroponics with Plant Growth-Promoting Microorganisms

**DOI:** 10.3389/fpls.2017.00674

**Published:** 2017-05-05

**Authors:** Roberta Paradiso, Carmen Arena, Veronica De Micco, Maria Giordano, Giovanna Aronne, Stefania De Pascale

**Affiliations:** ^1^Agricultural and Food Sciences, University of Naples Federico IINaples, Italy; ^2^Department of Biology, University of Naples Federico IINaples, Italy

**Keywords:** beneficial bacteria, chlorophyll fluorescence, controlled ecological life-support system (CELSS), *Glycine max* (L.) Merr., *Mycorrhizae*, nutrient film technique (NFT), stomata density, *Trichoderma* spp.

## Abstract

The use of hydroponic systems for cultivation in controlled climatic conditions and the selection of suitable genotypes for the specific environment help improving crop growth and yield. We hypothesized that plant performance in hydroponics could be further maximized by exploiting the action of plant growth-promoting organisms (PGPMs). However, the effects of PGPMs on plant physiology have been scarcely investigated in hydroponics. Within a series of experiments aimed to identify the best protocol for hydroponic cultivation of soybean [*Glycine max* (L.) Merr.], we evaluated the effects of a PGPMs mix, containing bacteria, yeasts, *mycorrhiza* and *trichoderma* beneficial species on leaf anatomy, photosynthetic activity and plant growth of soybean cv. ‘Pr91m10’ in closed nutrient film technique (NFT). Plants were grown in a growth chamber under semi-aseptic conditions and inoculated at seed, seedling and plant stages, and compared to non-inoculated (control) plants. Light and epi-fluorescence microscopy analyses showed that leaves of inoculated plants had higher density of smaller stomata (297 vs. 247 n/mm^2^), thicker palisade parenchyma (95.0 vs. 85.8 μm), and larger intercellular spaces in the mesophyll (57.5% vs. 52.2%), compared to non-inoculated plants. The modifications in leaf functional anatomical traits affected gas exchanges; in fact starting from the reproductive phase, the rate of leaf net photosynthesis (NP) was higher in inoculated compared to control plants (8.69 vs. 6.13 μmol CO_2_ m^-2^ s^-1^ at the beginning of flowering). These data are consistent with the better maximal PSII photochemical efficiency observed in inoculated plants (0.807 vs. 0.784 in control); conversely no difference in leaf chlorophyll content was found. The PGPM-induced changes in leaf structure and photosynthesis lead to an improvement of plant growth (+29.9% in plant leaf area) and seed yield (+36.9%) compared to control. Our results confirm that PGPMs may confer benefits in photosynthetic traits of soybean plants even in hydroponics (i.e., NFT), with positive effects on growth and seed production, prefiguring potential application of beneficial microorganisms in plant cultivation in hydroponics.

## Introduction

When properly managed, the hydroponic systems permit the optimal water and nutrient supply to the roots, helping to improve plant growth and yield and resource use efficiency compared to soil (Savvas et al., 2013; Paradiso et al., 2014b).

Recirculating hydroponic systems are used in most of the studies aiming to characterize plant production under controlled environment, in the sight of their use in CELSS (controlled ecological life-support system) in Space (Wheeler and Sager, 2003). Together with durum wheat, bread wheat and potato, soybean has been selected as a candidate crop for CELSS due to the high nutritional value of seeds (Palermo et al., 2012). To maximize crop performance, once crops have been chosen, the most suitable cultivar for CELSS has to be selected by considering plant adaptability to the hydroponic environment and other relevant agronomical requirements, such as small size, short growing cycle, high harvest index (as ratio of edible part to total biomass per plant), and good tolerance to biotic and abiotic stresses (De Micco et al., 2012).

It is conceivable that further improvements in crop productivity in hydroponics could be achieved by exploiting the action of beneficial organisms, known as plant growth-promoting organisms (PGPMs) (Lee and Lee, 2015). When grown in soil, plants normally establish specific interactions with PGPMs, which grow in, on, or around plant root tissues. These relationships are well characterized in the most important crop/microbe combinations in field (Hayat et al., 2010). PGPMs can promote plant growth and yield, directly or indirectly, through several mechanisms (Vessey, 2003; Mitter et al., 2013). Biological fixation of atmospheric N_2_, performed by specific strains of symbiotic *Rhizobia* bacteria in leguminous plants (Mylona et al., 1995), and non-symbiotic bacteria (e.g., *Azotobacter* spp., *Pseudomonas* spp.) in other crops (Kennedy et al., 2004), provides additional amount of N. Numerous bacteria [e.g., *Pseudomonas* spp., *Bacillus* spp., *Rhizobium* spp. (Rodríguez and Fraga, 1999)] and fungi [e.g., *Aspergillus* spp., *Penicillium* spp. (Whitelaw, 1999)] produce chelators able to convert insoluble minerals (e.g., phosphorus) to bioavailable forms, or to isolate heavy metals and toxic compounds, including pathogens metabolites. Some bacteria [e.g., *Pseudomonas* spp. (Luján et al., 2015)] and fungi [e.g., *Streptomyces, Actinomycetes* (Haas et al., 2008)] can solubilise ferric iron (Fe^3+^), by means of siderophores. Finally, many bacterial and fungal microorganisms produce phytohormones, such as indole-3-acetic acid (IAA), cytokinins, gibberellins, promoting plant growth (Weyens et al., 2009). Indirect positive effects of PGPMs on plant can be related to biocontrol mechanisms (Maksimov et al., 2011). For instance, competition for plant root exudates and mucilage, as source of nutrients, is a mechanism for pathogens exclusion (Compant et al., 2005). Siderophores produced by beneficial bacteria (e.g., *Pseudomonas*) have higher affinity to Fe^3+^ than those of pathogenic fungi (Beneduzi et al., 2012). Several PGPMs (e.g., *Pseudomonas* spp., *Bacillus* spp., *Trichoderma* spp.) produce antibiotic compounds and lytic enzymes which degrade pathogens cell walls and toxins (Maksimov et al., 2011); some others induce systemic resistance against pathogens through the mechanism of *priming* (Conrath et al., 2006), or help plants cope with abiotic stresses such as drought and salinity by means of various mechanisms (Yang et al., 2009).

In accordance with the positive effects exerted on plant growth, at the present the listed microorganisms are considered as plant biostimulants (Calvo et al., 2014), similarly to organic molecules from plant extracts, containing bioactive compounds able to activate plant metabolism improving plant performance (Bulgari et al., 2015).

Moreover, it is known that PGPMs can influence both gas exchanges and the whole photosynthetic machinery in several crops. In sugar beet (*Beta vulgaris* L.), some endophytic bacteria (e.g., *Bacillus pumilus*) enhanced the rate of photosynthesis and the maximal photochemical efficiency even at increasing Photosynthetic Photon Flux Density (PPFD), by promoting the chlorophyll synthesis and the electron transport in thylakoid membranes, and production of phytohormones, with positive effects on growth of both roots and aerial part (Shi et al., 2010). In runner bean (*Phaseolus coccineus* L.), the co-inoculation of seeds with two rhizobacteria strains, active for phosphorous solubilisation and production of siderophores (*Bacillus mycoides*) and indoleacetic acid (*Bacillus pumilus*), produced a synergistic action resulting, in increase in photosynthesis and chlorophyll content, particularly during vegetative and early flowering stages, and in the grain yield (Stefan et al., 2013). In plants of strawberry (*Fragaria* × *ananassa*), inoculation with arbuscular endomycorrhizal fungi (AMF) increased the photosynthetic and photochemical activity even under drought stress (Borkowska, 2002).

Most the listed effects of PGPMs have been well studied in soil, while little is known on these associations in hydroponics, where the benefits also depend on the ability of microbes to survive and proliferate over time, and to colonize plant roots in the specific environment (i.e., acid recirculating nutrient solution). Some PGPMs (*Pseudomonas* spp., *Bacillus* spp., endomycorrhizal fungi) have been successfully tested in hydroponically grown vegetables (e.g., tomato, cucumber, and lettuce), with positive effects on plant growth, yield and quality (Lee and Lee, 2015). However, most reports focus on the relief from biotic stress, demonstrating that growth promotion depended on disease suppression in recirculating systems, where pathogens can easily develop and spread, also considering that the absence of non-pathogenic competitor microorganisms increases their degree of danger (Peer and Schippers, 1989; Lee et al., 2010; Stewart-Wade, 2011).

Regarding soybean, positive effects of natural association or root inoculation with PGPMs have been well investigated in soil (Salvagiotti et al., 2008; Tewari et al., 2015). Conversely, a limited number of studies has addressed this topic for soybean in hydroponics, and they mainly focused on biological N-fixation (Vigue et al., 1977; Paradiso et al., 2014a, 2015).

Only a few works concern the impact of PGPMs on the host photosynthetic metabolism, which is crucial in determining plant growth and productivity, and they usually refer to single or double microbial species, while at the present mixed cultures are often preferred, since they match multiple scopes (e.g., increasing or restoring microbial diversity of soil, acting effectively in several plant species) (Lugtenberg et al., 2013).

In this study we aimed at analyzing the effects of inoculation with PGPMs on soybean leaf structure, which is fundamental in determining the photosynthetic performance and ultimately the plant growth and yield. Therefore, we investigated the effect of a commercial PGPMs mix on the photosynthetic activity and leaf anatomical functional traits of soybean cultivated in hydroponics under controlled environment.

## Materials and Methods

### Cultivation Design, Growth Chamber Environmental Control, and Hydroponic System Management

The study was conducted on plants of the soybean [*Glycine max* (L.) Merr.] cultivar (cv.) ‘PR91M10’ (Pioneer Intl.). This cultivar was previously selected according to the European Space Agency (ESA) criteria (De Micco et al., 2012) and evaluated in hydroponics in controlled environment (Paradiso et al., 2012; De Micco et al., 2013).

Plants were grown in a 28 m^2^ open-gas-exchange growth chamber (7.0 m × 2.1 m × 4.0 m, W × H × D), equipped with a computer for integrated climate control. The nutrient film technique (NFT) system consisted in polypropylene double gullies (each with two single gullies flanked). Each gully was 60 cm high, 100 cm long and 10 cm wide, had an inclination of about 1%, and was equipped with four sprinklers. Sixteen plants were grown per double gully and three double gullies (48 plants in total) were used per treatment. Gullies were sealed with white-on-black polyethylene film to prevent evaporation, to avoid the entrance of light in the root zone, and to enhance the light distribution in the basal part of the plants through reflection.

**FIGURE 1:**
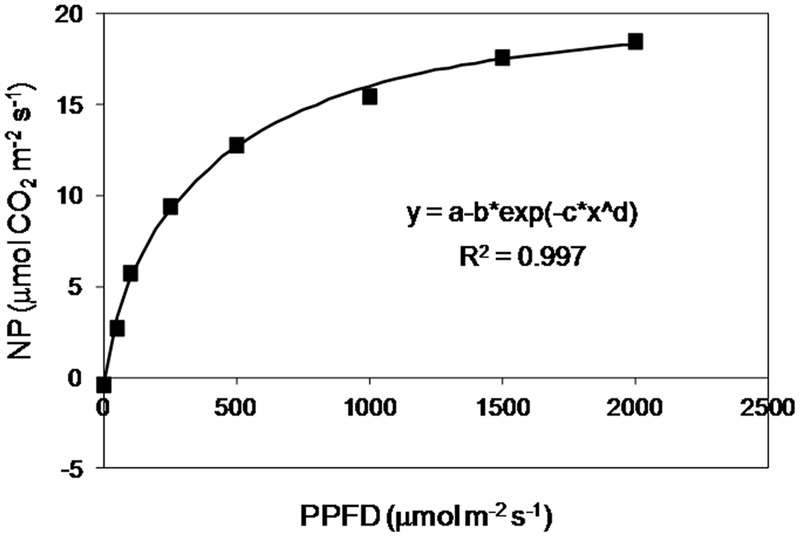
**Response curve of net photosynthesis (NP) to increasing light intensity in soybean cv. ‘PR91M10’ grown in closed-loop hydroponics.** Average values of three non-inoculated plants at vegetative phase; measurement conditions: 26°C, RH 70%, CO_2_ 400 ppm.

Before starting the experiment, the selected cv. ‘PR91M10’ was characterized for photosynthetic response to light intensity, performing a light fast kinetics curve (**Figure 1**), to identify the most suitable value of PPFD for photosynthesis to be applied in closed controlled environment. Based on the curve, the light intensity in the growth chamber was set at 420 μmol m^-2^ s^-1^ at the top of the canopy avoiding an excess of light and photoinhibitory damage risks to photosynthetic apparatus (Evans et al., 1993). Light was provided by High Pressure Sodium (HPS) lamps. Lamps were mounted on a mobile panel, which was moved upward following the stem elongation, in order to keep the PPFD constant at the canopy level, according to a day/night regime of 12/12 h. The other climatic parameters were chosen according to the Space-related literature on soybean (Dougher and Bugbee, 1997; Wheeler et al., 2008), and kept constant during the entire growth cycle. Temperature was 26/20°C (light/dark) and relative humidity (RH) was 70–80%. The mean values of temperature and RH recorded at the end of the experiment (98 days) were 26.3 ± 0.1/19.7 ± 0.1°C and 82.4 ± 2.9/68.7 ± 0.5%, respectively (Mean Value ± Standard Deviation). Cultivation was carried out at ambient CO_2_ concentration (370–400 ppm).

Each double gully was equipped with a polypropylene reservoir (21 liters) for the recirculating nutrient solution, and its own submerged pump (New A Jet 3000) in order to work independently. Nutrient solution was pumped from the tank into the gullies at a flow rate of 2.0 L/min and returned to the reservoir by gravity dependent flow. Fertigation started 14 days after sowing (DAS), and was performed continuously.

**FIGURE 2:**
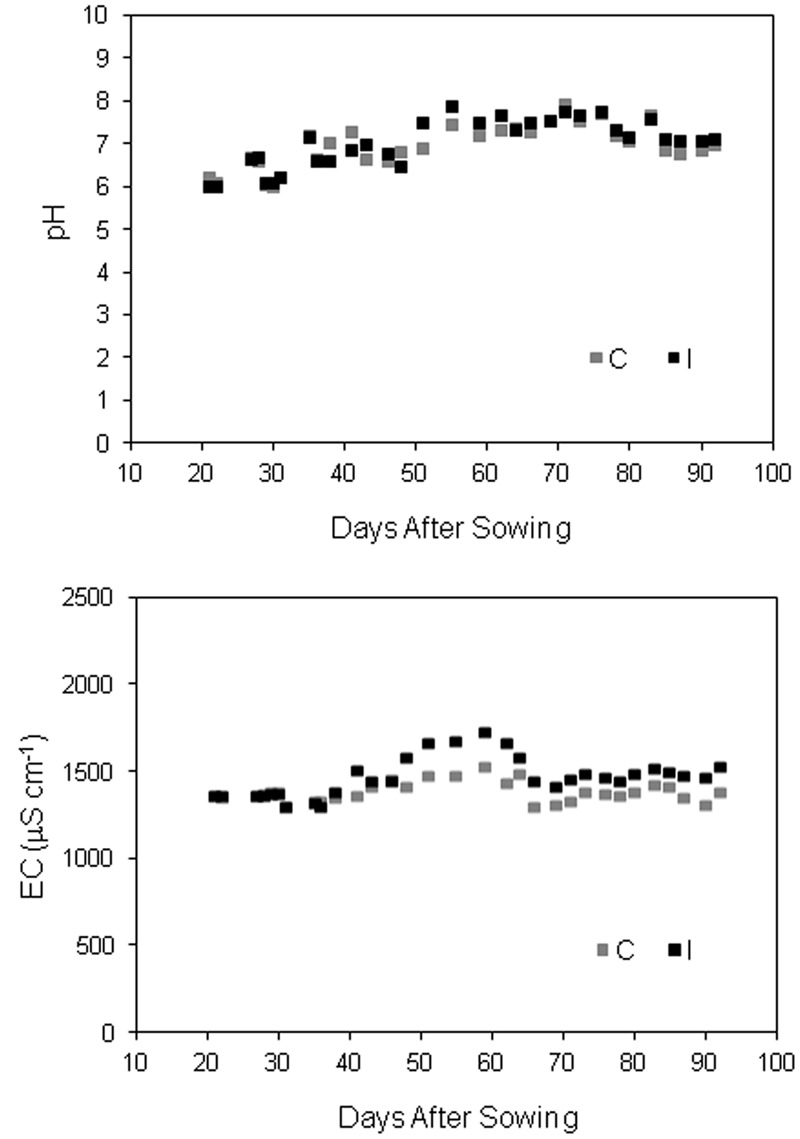
**Evolution of pH and EC of recirculating nutrient solution in soybean cv. ‘PR91M10’ grown in closed-loop hydroponics, before the adjustment to the target values (pH 5.8; EC 1200 μS cm^-1^ in control and 1400 μS cm^-1^ in inoculated treatment)**.

The nutrient solution was based on the standard Hoagland recipe 1/2 strength (Hoagland and Arnon, 1950), modified by Wheeler et al. (2008), according to the specific requirements of soybean. The starting nutrient solution had the following element concentration: 7.5 mM N, 3.0 mM K, 0.5 mM P, 2.5 mM Ca, 1.0 mM Mg, 1.0 mM S, 60 μM Fe, 7.4 μM Mn, 0.96 μM Zn, 1.04 μM Cu, 7.13 μM B, and 0.01 μM Mo. P content was reduced to 0.25 mM during the first 3 weeks of cultivation to avoid negative effects on mycorrhiza in the inoculated treatment (Colla et al., 2008). The same reduction was applied in control plants. EC and pH of the recirculation nutrient solution were controlled manually (Multimeter Basic 30, Crison Instruments, Barcelona, Spain) and adjusted every 2 days to the target values by adding deionised water and/or fresh solution (for EC control) and 1M nitric acid (for pH control) in the storage tank (**Figure 2**). The pH was kept at 5.8 in both the treatments. Since the addition of the inoculum increased the EC value in the fresh solution compared to control, EC target was raised from 1200 μS cm^-1^ in control to 1400 μS cm^-1^ in inoculated treatment. The starting and the replenish solution and deionised water were filtered at 0.45 mm. To prevent large fluctuation in the anions/cations concentrations, electrical conductivity and pH, the nutrient solution in both the treatments was renewed in all tanks 32 DAS.

### Disinfection Procedures and Seed Inoculation Protocol

Before starting cultivation, a disinfection of the growth chamber (floor, walls, gullies, conditioning devices, etc.) was performed with sodium hypochlorite water solution (NaClO 5 g l^-1^). Safety procedures were adopted during the experiment to minimize contamination from operators. In addition, periodic cleaning was performed with NaClO water solutions (5 g l^-1^ and 1 g l^-1^, for chamber and measurement tools, respectively).

Prior to start the experiment, the germination performance of cv. ‘PR91M10’ were evaluated at 8 DAS, according to the International Rules for Seed Testing (International Seed Testing Association, 1999). The Mean Germination Time (MGT) was calculated according to the following formula: MGT = Σ Dn/Σn, where *n* = number of seeds germinated per day, *D* = number of days from the beginning of the test. The germination percentage and MGT were 98.0 ± 2.4% and 4.5 ± 0.2 days, respectively, and were not affected by seed inoculation.

Seed sterilization was performed according to the protocol of Somasegaran and Hoben (1994). Seeds were rinsed in 95% ethanol for 20 s to remove waxy materials, then they were completely submerged in a NaClO water solution (2.5%) and gently swirled for 5 min. Sodium hypochlorite was drained off and seeds were rinsed six times in sterile H_2_O.

After sterilization, seeds were inoculated by submersion in a solution of Myco Madness mix (Humboldt Nutrients, Eureka, CA, USA), containing a mixture of 14 bacteria, yeasts and 12 beneficial fungi species (*Mycorrhizae* and *Trichoderma* spp.). The Myco Madness mix was chosen because of the high diversity of potential beneficial microbes.

The inoculum was prepared by adding 0.5 g of Myco Madness powder to 1 L of sterile quarter-strength Ringer solution (Oxoid, Milan, Italy). The cell count to verify the final concentration of the inoculum was performed by using a Thoma cell counting chamber (depth 0.02 mm, area 1/400 mm^2^; Thoma, Hawksley, United Kingdom) and the microscope Eclipse E200 Nikon, and resulted 5 × 10^5^ cells per ml. Incubation was performed for 12 h, at room temperature in the darkness (Bashan, 1986). Seeds of control treatment were dipped in sterile quarter-strength Ringer solution only, and submitted to similar conditions.

For germination, seeds were placed on three sterilized layers of filter paper (Watman n. 1) moistened with the inoculum (treated seeds) or with the quarter-strength Ringer solution only (control seeds), in Petri dishes (20 seeds per dish, 10 dishes per treatment). Dishes were sealed with parafilm and incubated at 22°C, in the darkness (Fernandez-Orozco et al., 2008). After 8 DAS, seedlings were transferred into sterilized rockwool plugs and moved in the hydroponic gullies, in the growth chamber. Inoculation was repeated on seedlings at transplanting (12 DAS), by submerging the roots in the Myco Madness solution for 24 h. During cultivation, inoculation was repeated by adding the inoculum to the recirculating nutrient solution, three times at 10-day interval starting from 38 DAS. Details on inoculum, inoculation protocol and rhizosphere microbiology are reported by Sheridan et al. (2016).

### Sampling and Measurements

The effects of the treatments were evaluated on plants in terms of changes in leaf structure, photosynthesis, and plant growth and productivity, also unraveling whether changes in photosynthesis are linked with modifications in leaf functional anatomical traits and photochemistry.

#### Functional Anatomical Traits of Leaves

Sampling for anatomical analyses was done at 57 DAS on the 3th fully expanded trifoliate leaf from the top of the plant. More specifically, 3 middle leaflets from 3 plants per treatment were cut and immediately submerged in the chemical fixative FAA (40% formaldehyde – glacial acetic acid – 50% ethanol, 5:5:90 by volume). After 2 weeks of fixation, leaflets were halved under a dissection microscope (SZX16, Olympus, Germany) to obtain two twin groups of subsamples: one devoted to the quantification of stomata traits, the other to the analysis of lamina cross sections.

For the analysis of stomata, three strips of lamina adaxial epidermis were peeled off from each subsample and mounted on microscope slides with distilled water. Epidermal peels were analyzed under a transmitted light microscope (BX60, Olympus) and digital images were collected by means of a digital camera (CAMEDIA C4040; Olympus), avoiding main veins. Digital images were analyzed through the software program Analysis 3.2 software (Olympus) to quantify stomata frequency and size. More specifically, stomata frequency was expressed as number of stomata per surface unit (mm^2^), counted in two regions per peel. Stomata size was quantified by measuring the length of at least 15 guard-cells (pole to pole) and the width of the same cells in the median position.

To obtain cross sections of the leaf lamina, the subsamples were cut under the SZX16 dissection microscope to obtain subsamples of 5 mm × 5 mm from the median part of the leaflet, avoiding the main vein. These subsamples were dehydrated in an ethanol series (up to 90%) and embedded in the acrylic resin JB4 (Polysciences, Warrington, PA, USA). Thin cross sections (5 μm) were cut by means of a rotary microtome, stained with 0.025% Toluidine blue in 0.1 M citrate buffer at pH 4 (Reale et al., 2012) and mounted with Canadian Balsam. The sections were analyzed under a BX60 light microscope and digital images were collected at different magnifications. By means of the Olympus Analysis 3.2 software, the mesophyll was characterized by measuring the thickness of palisade and spongy parenchyma tissues and the quantity of intercellular spaces in the spongy tissues. The thickness of palisade and spongy tissues were measured in five positions along the lamina, avoiding veins. The incidence of intercellular spaces was measured as the percentage of tissue occupied by intercellular spaces over a given surface, in six regions along the leaf lamina.

#### Photosynthesis, Fluorescence Measurements, and Chlorophyll Content

The light fast kinetics curve was performed at a single leaf level on non-inoculated plants, using a portable Infra Red Gas Analyzer WALZ HCM 1000 (Walz, Effeltrich, Germany) (**Supplementary Figure S1**). The curve was determined on the middle leaflet of the 2nd and 3th fully expanded trifoliate leaves from the top of the plant (two leaves per plant, three plants). Increasing PPFDs (0, 50, 100, 250, 500, 1000, 1500, and 2000 μmol m^-2^ s^-1^) were obtained by using a built-in halogen lamp, and the conditions inside the leaf chamber were kept constant: temperature 25°C, RH 70%, ambient CO_2_ concentration.

During plant cultivation, NP was measured on the same leaf types chosen for the light response measurements (two leaves per plant, three plants per treatment), at ambient light intensity (420 μmol m^-2^ s^-1^) and the same leaf chamber conditions. Measurements were carried out in the different phenological phases: vegetative growth (30 DAS), flowering (44 DAS), pod setting (57 DAS). NP was not detectable during the pod filling, because of the difficulty to position the leaves in the leaf chamber in presence of symptoms of senescence (wilting and curling).

On the same leaves, chlorophyll *a* fluorescence were determined using a portable FluorPen FP100 max fluorometer (**Figure 2**), equipped with a light sensor (Photon System Instruments, Brno, Czech Republic), at room temperature (26°C). The ground fluorescence *F*_o_ was induced on 30′ dark adapted leaves, by a blue LED internal light of about 1–2 μmol photons m^-2^ s^-1^. The maximal fluorescence level in the dark *F*_m_ was induced by 1 s saturating light pulse of 5.000 μmol photons m^-2^ s^-1^. The maximum quantum efficiency of PSII photochemistry was calculated as (*F*_m_-*F*_o_)/*F*_m_. The measurements in the light were conducted at PPFD of 420 μmol (photons) m^-2^ s^-1^ at the canopy level. The PSII quantum yield (QY) was determined by means of an open leaf-clip suitable for measurements under ambient light, according to Genty et al. (1989). QY was used to calculate the linear electron transport rate (ETR), according to Krall and Edwards (1992). Non-photochemical quenching (NPQ) was calculated as described by Bilger and Björkman (1990), according to the following formula: NPQ = (*F*_m_/F_m_′)-1. Measurements started at flowering (44 DAS), as significant differences in NP between the treatments were detected, and repeated during pod setting (57 DAS) and pod filling (84 DAS).

After fluorescence determinations, the leaf greenness was estimated using a colorimeter (Chlorophyll Meter Konica-Minolta SPAD 502), and expressed as SPAD units, in six plants per treatment (two leaves per plant, five measurements per leaf), at flowering (44 DAS). Measurements were made at the central point of the leaflet between the midrib and the leaf margin. In the same samples, chlorophyll *a* and *b* content was determined by extraction in acetone and spectrophotometer lecture (Jeffrey and Humphrey, 1975), using a Hach DR 4000 Spectrophotometer (Hach Company, Loveland, CO) on one 2-cm^2^ leaf sample per leaf.

#### Plant Growth and Productivity, and Chemical Analyses

Growth analysis during the growing cycle was based on non-destructive measurements of plant height and leaf number, determined every 7 to 10 days, on six plants per treatment. Plant leaf area (LA) was estimated by the values of leaf length and width, using the formula of Wiersma and Bailey (1957), based on the specific soybean leaf types and shapes.

Soybean seeds were harvested when pods had turned brown (average water content 14%). At each harvest, yield data [fresh weight (FW) of pods and seeds] were determined per single plant. Plant productivity was measured as grams of seeds per plant^-1^ (edible biomass).

At the end of the experiment, plants were collected to determine FW and dry matter (DM), and their partitioning in roots, stems and leaves (non-edible biomass). Measurements were carried out on six plants per treatment (two plants × double gully). DM was measured after oven-drying at 60°C until constant weight.

The concentration of the main cations (K^+^, Ca^2+^, Mg^2+^) and anions (NO_3_^-^) in the recirculating nutrient solution and in the leaf tissues was determined using an ion exchange chromatographer (ICS-3000, Dionex, Sunnyvale, CA, USA). Nutrient solution samples were collected at 7-day intervals, starting from the first inoculation of the recirculating solution. Leaf analysis was performed on water extract of DM of 5 healthy, fully expanded leaves per treatment randomly sampled, during the flowering phase.

### Statistical Analysis

All data were processed with one-way ANOVA (*p* < 0.05), using the SPSS^®^statistical package (SPSS Inc., Chicago, IL, USA). Shapiro–Wilk and Kolmogorov–Smirnov Tests were performed to check for normality. Percent data were transformed through arcsine function before statistical analysis.

## Results

### Leaf Functional Anatomical Traits

**FIGURE 3:**
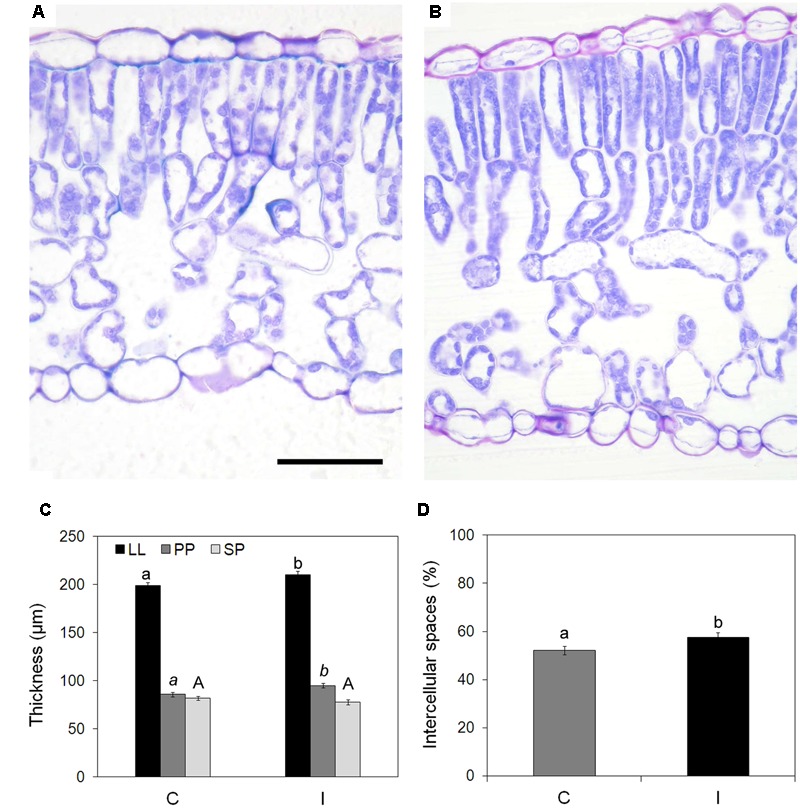
**Effect of root inoculation on mesophyll traits in leaves of soybean cv. ‘PR91M10’ grown in closed-loop hydroponics.** Light microscopy views of cross sections of leaves from control **(A)** and inoculated **(B)** plants. Bar = 50 mm (images are at the same magnification). Thickness of leaf lamina (LL), palisade parenchyma (PP), and spongy parenchyma (SP) **(C)**, and percent of intercellular spaces **(D)** measured in control and inoculated plants (Mean ± standard error, *n* = 45). Different letters indicate significant differences at *P* < 0.05. In **(C)**, small letters refer to LL, italic small letters refer to PP, capital letters refer to SP.

Analyzed leaves showed a typical dorsiventral structure (**Figures 3A,B**), with mesophyll made of two layers of palisade cells and a spongy parenchyma rich in intercellular spaces. Stomata were frequent on adaxial epidermis, while rare on abaxial surface.

Root inoculation was responsible for a significant increase in leaf lamina thickness, due to the palisade parenchyma that was thicker in inoculated than in control plants (95.0 vs. 85.8 μm) (**Figure 3C**). Leaves of inoculated plants also showed a more loosen spongy parenchyma because of a higher percentage of intercellular spaces compared with leaves from non-inoculated plants (57.5% vs. 52.2%) (**Figure 3D**).

**FIGURE 4:**
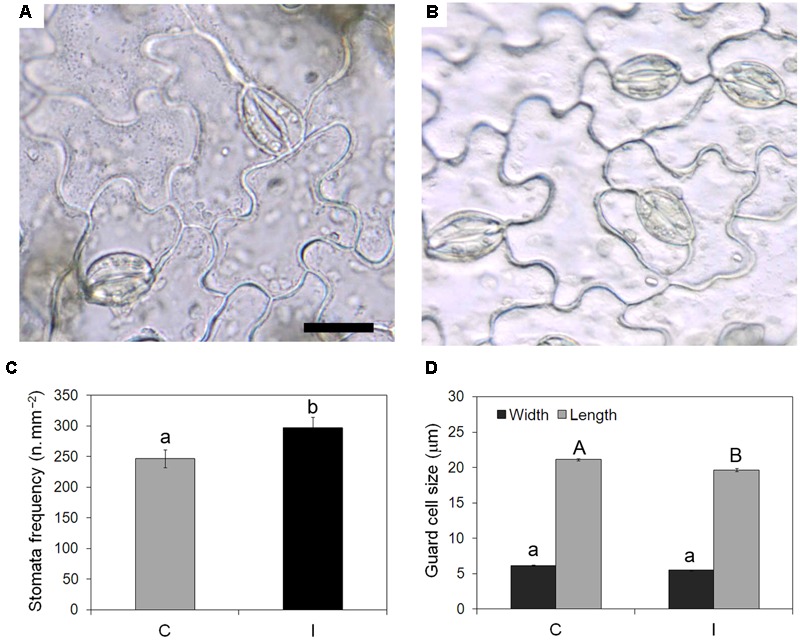
**Effect of root inoculation on stomata traits in leaves of soybean cv. ‘PR91M10’ grown in closed-loop hydroponics.** Light microscopy views of epidermal peels showing stomata in leaves from control **(A)** and inoculated **(B)** plants. Bar = 20 mm (images are at the same magnification). Stomata frequency **(C)** and size **(D)** quantified in control and inoculated plants (Mean ± standard error, *n* = 54 for stomata frequency, *n* = 135 for stomata size). Different letters indicate significant differences at *P* < 0.05. In **(D)**, small letters refer to guard cell width, capital letters refer to guard cell length.

Leaves of inoculated plants showed significantly higher stomata frequency (297 vs. 247 n/mm^2^) (**Figures 4A–C**) than non-inoculated controls. Stomata were significantly smaller in leaves of inoculated plants compared to the control ones, due to reduced length and width of guard cells (**Figure 4D**).

### Photosynthesis, Fluorescence Measurements, and Chlorophyll Content

**FIGURE 5:**
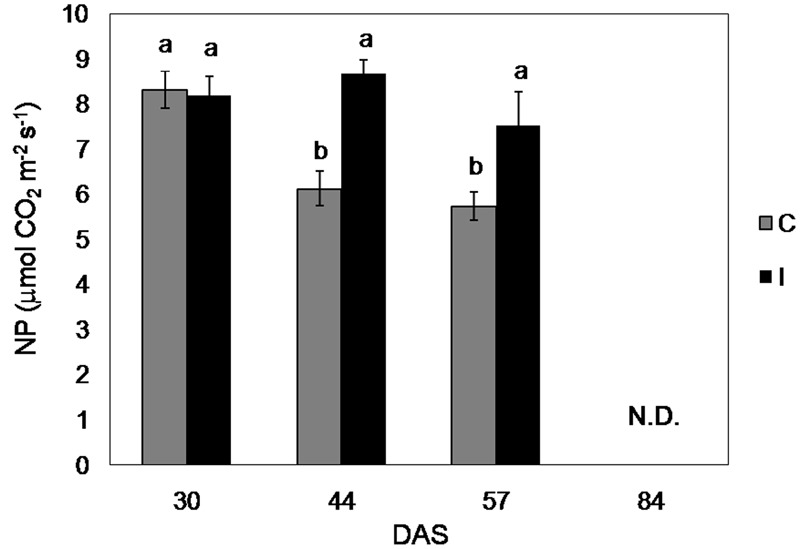
**Net photosynthesis of soybean cv. ‘PR91M10’ in control and inoculated plants grown in closed-loop hydroponics, throughout the developmental cycle: vegetative growth (30 DAS), flowering (44 DAS), and fruit setting (57 DAS).** (Means ± standard error; *n* = 3). Within each date, different letters indicate significant differences at *P* < 0.05.

Net photosynthesis of fully developed leaves of soybean control plants was maximum in the vegetative phase and decreased progressively during the reproductive phase, ranging from 8.3 μmol CO_2_ m^-2^ s^-1^ (30 days after sowing, DAS) to 5.7 μmol CO_2_ m^-2^ s^-1^ (57 DAS) (**Figure 5**). Measurements of NP throughout the growing cycle did not show significant differences between the treatments during the vegetative phase (until 30 DAS), while they revealed higher CO_2_ assimilation in inoculated plants from the flowering (**Figure 4**). Specifically, NP in inoculated plants was 42 and 31% higher compared to the control, at 44 and 57 DAS, respectively.

**FIGURE 6:**
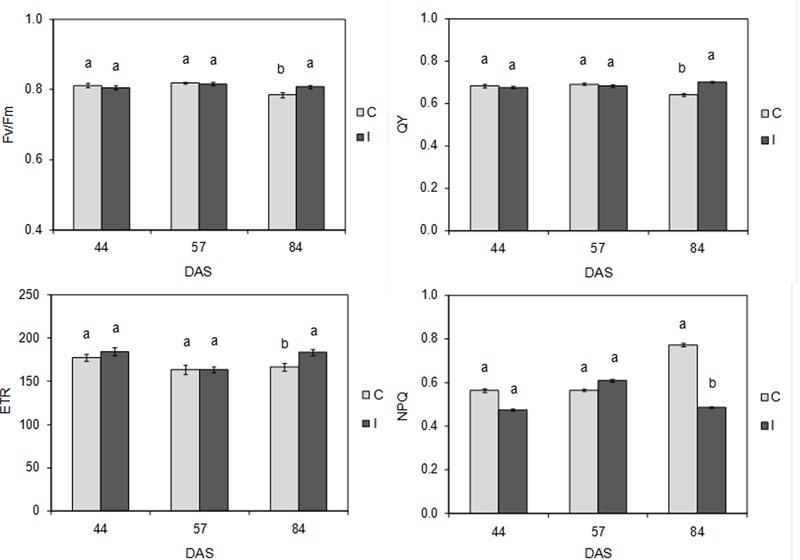
**Maximal PSII photochemical efficiency (*F*_v_/*F*_m_), PSII quantum yield (QY), linear electron transport rate (ETR), and non-photochemical quenching (NPQ) in control and inoculated plants of soybean cv. ‘PR91M10’ grown in closed-loop hydroponics, throughout the developmental cycle: flowering (44 DAS), fruit setting (57 DAS) and pod filling (84 DAS).** (Mean ± standard error; *n* = 18). Within each date, different letters indicate significant differences at *P* < 0.05.

Root inoculation did not determine any significant effects on plant photochemistry at 44 and 57 DAS, as demonstrated by the values of maximal PSII photochemical efficiency (*F*_v_/*F*_m_), PSII quantum yield (QY), linear electron transport rate (ETR), and non-photochemical quenching (NPQ), which were not significantly different between control and inoculated plants (**Figure 6**). Conversely, at 84 DAS, significant differences were detected in the efficiency of light conversion to reaction centers. In particular, compared to control, treated plants showed a significant (*P* < 0.05) increase in *F*_v_/*F*_m_ (0.807 vs. 0.784), QY (0.702 vs. 0.640) and ETR (183 vs. 166), as well as a significant (*P* < 0.01) reduction in NPQ (0.486 vs. 0.773).

The higher photochemical efficiency in inoculated plants did not match the chlorophyll content. In fact, the comparison between inoculated and non-inoculated plants showed no difference between treatments in the leaf chlorophyll content, neither when determined as chl *a* and chl *b* concentration, nor when measured as greenness (**Table 1**).

**Table 1:** Leaf greenness estimated using a colorimeter (Chlorophyll Meter Konica-Minolta SPAD 502), and chlorophyll *a* and *b* content determined by extraction in acetone and spectrophotometer lecture in plants of soybean cv. ‘PR91M10’ grown in closed-loop hydroponics (vegetative phase; *n* = 6).

	SPAD (SPAD units)	Chl a (mg ml^-1^)	Chl b (mg ml^-1^)	Chl a/Chl b (ratio)	Tot Chl (mg ml^-1^)
C	33.33	6.94	1.72	4.0	8.66
I	34.33	6.78	1.64	4.1	8.42
	ns	ns	ns	ns	ns

### Hydroponic System Management, Plant Growth and Yield, and Leaf Chemical Analyses

The evolution of pH and EC in the recirculating nutrient solution is shown in **Figure 2**, for both control and inoculated gullies. The pH value after 2 days of recycling was always higher than in the fresh or adjusted solution (target value 5.8). The fluctuations were smaller during the 1st weeks of cultivation, when the plant size was still small, while they became wider as plant developed, and water and nutrient uptake increased. Accordingly, EC variations were very narrow in the first 30 days, increased during the vegetative growth and decreased progressively after the beginning of leaf falling (around day 65). On the average of the entire experiment, the value was similar between the treatments for pH (6.97 on average), while it was higher in inoculated compared to control gullies for EC (1465 ± 21 vs. 1381 ± 10 μS cm^-1^; Means ± Standard error of 32 measurements).

The concentration of the main nutrients (in mmol l^-1^) in the recirculating nutrient solution was not significantly different between control and inoculated treatment for N (7.79 ± 0.59 vs. 7.58 ± 0.74), K (2.92 ± 0.14 vs. 3.38 ± 0.16), Ca (3.04 ± 0.09 in C vs. 2.90 ± 0.09), Mg (0.61 ± 0.09 vs. 0.58 ± 0.11) (Mean ± Standard error; *n* = 6).

The growing cycle of soybean ‘PR91M10’ in closed-loop NFT under controlled environment lasted 98 days, from the sowing to the end of the harvests, in both inoculated and non-inoculated treatments.

**FIGURE 7:**
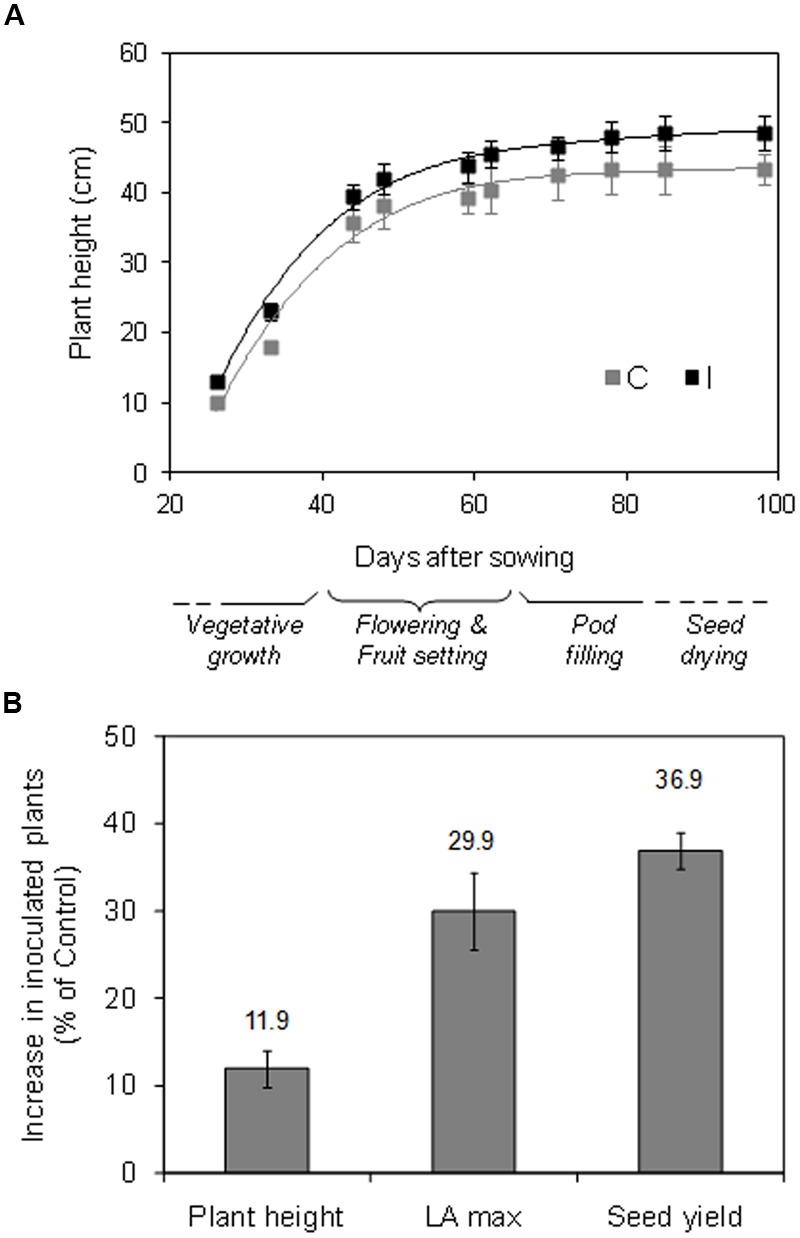
**Time evolution of plant height and sequence of the phenological phases in the growth cycle (A)**, and percentage increase in final plant height, maximum leaf area (LA, before leaf shedding), and seed yield **(B)** in inoculated vs. non-inoculated plants, in soybean cv. ‘PR91M10’ grown in closed-loop hydroponics (mean values ± standard Error; *n* = 6).

In control plants, flowering started around 34 DAS, and it was followed by the pod setting (until 60 DAS) and the filling and drying of pods and seeds, until the harvest, which started at 88 DAS and lasted 10 days (**Figure 7A**). Root inoculation did not affect significantly the earliness and the duration of the different phenological stages, as well as the harvesting time.

Inoculation promoted an increase, although not significant, in root growth (+10.9% on a DM basis – data not shown), while it significantly improved the growth of the aerial part and the seed production (**Figures 7A,B**). Specifically, during the whole experiment, inoculated plants showed a tendency to higher values of plant height, and were significantly taller at the harvest compared to control (48.5 vs. 43.3 cm) (**Figure 7A**). Accordingly, the total plant leaf area (maximum value before leaf shedding) and the seed yield were higher in inoculated compared to control plants (+29.9 and +36.9%, respectively) (**Figure 7B**).

Chemical analysis revealed changes in composition of leaf tissues at the stage of flowering. Specifically, inoculated plants showed a tendency to higher values compared to control in the content (g per 100 g D.M.) of NO_3_ (0.15 ± 0.04 vs. 0.11 ± 0.01), K (2.40 ± 0.16 vs. 2.18 ± 0.12), Ca (1.28 ± 0.18 vs. 1.03 ± 0.13), even if the difference between the treatments was found to be significant only for Mg (0.20 ± 0.04 vs. 0.13 ± 0.01).

## Discussion

The strategy of nutrient solution control adopted in the experiment, with measurements and adjustments at 2-day intervals, was efficient in containing the EC and pH fluctuations within acceptable values, and to guarantee comparable nutrients supply in the control and inoculated treatments.

Root inoculation with the Myco Madness mix, containing bacteria, yeasts, *mycorrhiza* and *trichoderma* beneficial species, promoted the overall plant growth and the seed production of soybean cv. ‘PR91M10,’ grown in NFT under controlled environment.

It is known that the action of most PGPMs is based on the combination of two or more modes of action, as described by the “additive hypothesis” (Bashan and Holguin, 1997); moreover, synergistic effects are reported in numerous microorganisms when co-inoculated. Indeed, the promotion of plant growth observed in soybean inoculated with PGPMs in our experiment was presumably related to multiple mechanisms (Mitter et al., 2013).

The time course of NP of ‘PR91M10’ plants in hydroponics followed a normal pattern for soybean, with declining rates from the vegetative to the reproductive phase, reflecting the plant aging and the consequent leaf senescence (Paradiso et al., 2015). However, inoculation determined significantly higher photosynthetic rates starting from the flowering, and a slower decrease during plant maturity and senescence compared to control. In soybean, two metabolic changes which reduce the efficiency of the photosynthetic machinery over time are documented: the mobilization of nutrients (especially N) from leaves to developing seeds (which directly lowers photosynthetic output), and the decreasing in root growth and functioning, which slows the xylem flow of water and nutrients and the hormonal translocation (indirectly restricting the rate of photosynthesis) (Imsande, 1988). In our experiment, it is conceivable that the severity of both these processes was alleviated in inoculated plants. Indeed, inoculation seemed to promote the growth of root system, likely enhancing the capability of nutrient uptake. In addition, several microorganisms colonizing the rhizosphere are known to improve plant nutrition and to produce phytohormones, that could have counterbalanced the detrimental effect of the root aging on mineral and hormonal transport (Ahemad and Kibret, 2014). As matter of fact, at the end of our experiment the microbial characterization revealed that the rhizoplane/endosphere of inoculated soybean plants were strongly dominated by *Ochrobactrum* bacteria (Sheridan et al., 2016), which is a plant growth-promoting *taxon*, known to exhibit a wide range of positive actions on plant nutrition and hormonal balance (Imran et al., 2014, 2015; Muangthong et al., 2015). Increase of photosynthetic rate was also previously ascribed to selected strains of *Ochrobactrum*, even in presence of soil waterlogging stress, where it reduced the plant ethylene production (Grichko and Glick, 2001; Barnawal et al., 2012), and to several Bacilli (Wu et al., 2016).

Our data on chlorophyll *a* fluorescence revealed that the increase of photosynthetic rate and photochemistry in inoculated plants was not due to higher light harvesting capacity, since the total chlorophyll content did not change compared to the control. Instead, it is conceivable that inoculated plants were able to use and convert light more efficiently to photosystems, reducing the occurrence of photoinhibitory damage risks (Shi et al., 2010). In accordance with this hypothesis, photochemistry analysis showed that the two groups of plants regulated differently the light utilization to reaction centers: more specifically, inoculation reduced the dissipation of light energy as heat, promoting the electron transport rate to C fixation; this allowed to allocate the most part of reductive power in carbon assimilation process, enhancing plant biomass accumulation. Conversely, the control plants, that exhibit low photochemical efficiency, needed to dissipate thermally the excess of absorbed light within photosystems, in order to avoid photoinhibition (Maxwell and Johnson, 2000). This strategy is effective in guarantying the integrity of photosystems but it reduces the plant efficiency to assimilate CO_2_ and to accumulate biomass. The success of a such regulation is demonstrated by the values of maximum photochemical efficiency (*F*_v_/*F*_m_) that are comparable in both the plant groups, indicating the absence of injuries to photosynthetic apparatus regardless of the treatment. These evidences are consistent with the findings of Shi et al. (2010) who found in sugar beet an increase of gas exchanges and photochemistry triggered by some endophytic bacteria (e.g., *Bacillus pumilus*). Accordingly, in our experiment, some Bacilli (*Staphylococcus* spp.) were found in the root exosphere of inoculated plants, together with other beneficial bacterial *taxa* such as Actinobacteria, Betaproteobacteria, and Alphaproteobacteria (Sheridan et al., 2016), known to exert several useful effects on plant metabolism (Ventorino et al., 2014; Chauhan et al., 2015; Castellano-Hinojosa et al., 2016). Moreover, the higher photosynthetic rate could be likely ascribed not only to a more efficient photochemistry, but also to an improvement of Rubisco carboxylation capacity, hypothesized on the basis of the enhancement of gas exchanges in inoculated plants.

In addition to the effect on photochemistry, inoculation enhanced photosynthesis even by inducing changes in leaf anatomical traits. The higher stomata frequency found in treated plants could be interpreted as a plant strategy to satisfy the increasing demand of CO_2_ needed to match the higher growth rate in inoculated plants. Beside, higher stomata density can greatly amplify the potential for control over water loss rate and CO_2_ uptake. Moreover, the occurrence of smaller stomata in inoculated plants compared to non-inoculated ones would allow better control of stomata opening/closure since small stomata are responsible for faster dynamic characteristics (Drake et al., 2013). Indeed, in our experiment, inoculation induced the formation of leaves whose structural traits can support more dynamic regulation of stomata opening/closure.

The presence of high frequency of small stomata is known to have a direct positive influence on the operating stomata conductance which in turn scaled with leaf gas-exchange (Meinzer, 2003; Barbieri et al., 2012): leaves with small and numerous stomata are considered capable of attain high or low stomata conductance when environmental conditions are, respectively, favorable or unfavorable (Drake et al., 2013). Moreover, the number and size of stomata are also related to plant transpiration balance (Meinzer, 2003). A strong stomata control may allow using the same amount of water more efficiently by root apparatus of inoculated plants. Generally a high water use efficiency is obtained limiting gas exchanges through the increase of stomata closure. In the case of inoculated plants, the elevated number of stomata and their reduced size allow to maintain more stomata opened on the leaf lamina in order to favor CO_2_ entrance in substomatal spaces and at same time to minimize the water vapor losses. From this point of view the PGPMs stimulating the evolution of some specific anatomical traits (i.e., increase on intercellular spaces, elevated number of stomata) may have also indirectly affected plant-substrate water relationships, and consequently the nutrient and water uptake (Balliu et al., 2015).

The relations between operating stomata conductance and the physical attributes of stomata has been shown both on a large evolutionary scale and on a smaller scale in response to specific environmental conditions (Hetherington and Woodward, 2003; Franks and Beerling, 2009). Plant photosynthetic productivity and water-use efficiency are linked not only to stomatal conductance but also to mesophyll resistance, thus to leaf anatomy (Brodribb et al., 2007; Woodruff et al., 2009). More specifically, traits such as thickness of palisade and spongy parenchyma and their porosity, affect the lateral and vertical gas diffusion within the leaf lamina (Pieruschka et al., 2005). In inoculated plants, the thicker leaf lamina would not increase mesophyll resistance compared to non-inoculated plants, because it is accompanied by increased intercellular spaces which would improve the accessibility to the carboxylation sites of the chloroplasts inside the cells. Moreover, the improved photosynthesis in inoculated plants is in line with the increased thickness of palisade parenchyma which contain most of the chloroplasts.

## Conclusion

Root inoculation with the Myco Madness microbial mix, containing bacteria, yeasts, *mycorrhiza* and *trichoderma* beneficial species, enhanced the photosynthetic activity of soybean ‘Pr91M10’ grown in closed-loop NFT. Starting from flowering, the rate of leaf NP was higher in inoculated plants compared to controls. This result was found to be related to changes in leaf functional anatomical traits promoting leaf gas exchanges: leaves of inoculated plants showed higher density of smaller stomata, a thicker palisade parenchyma, and larger intercellular spaces in the mesophyll, compared to non-inoculated plants. In addition, inoculation determined higher photochemical efficiency in adult plants, during the stage of seed maturation, thanks to the higher efficiency to use and convert light to photosystems.

Overall, the positive influence of PGPMs root inoculation on leaf photosynthetic performances enhanced plant growth and seed production of soybean grown hydroponically.

In conclusion, in our experimental conditions, inoculation with PGPMs conferred benefits in photosynthesis and leaf functional anatomical traits, which in turn enhanced plant growth and productivity of soybean grown in closed-loop hydroponics under controlled environment. These results prefigure potential application of beneficial microorganisms in hydroponic cultivation of plants.

## Author Contributions

RP and MG performed plant cultivation. RP, CA, and VDM performed measurements, data collection and statistical analysis, and wrote the manuscript. GA and SDP provided scientific oversight in experimental design and interpretations and contributed in writing the manuscript. SDP obtained funding for the study.

## Conflict of Interest Statement

The authors declare that the research was conducted in the absence of any commercial or financial relationships that could be construed as a potential conflict of interest.

## Supplementary Material

FIGURE S1 **Plants of soybean cv. ‘PR91M10’ grown in closed-loop NFT (A)**. Measurements of photosynthesis with the Infra Red Gas Analyzer WALZ HCM 1000: particular of the halogen lamp for light response curves **(B)**, and measurement at the ambient light intensity **(C)**. Measurements of chlorophyll *a* fluorescence with the FluorPen FP100 max fluorometer **(D)**.
