# Supplementary figures and images for: Changes in Leaf Anatomical Traits Enhanced Photosynthetic Activity of Soybean Grown in Hydroponics with Plant Growth-Promoting Microorganisms
Source: Front Plant Sci. 2017 May 5;8:674. doi: 10.3389/fpls.2017.00674 (PMC5418343; doi:10.3389/fpls.2017.00674)

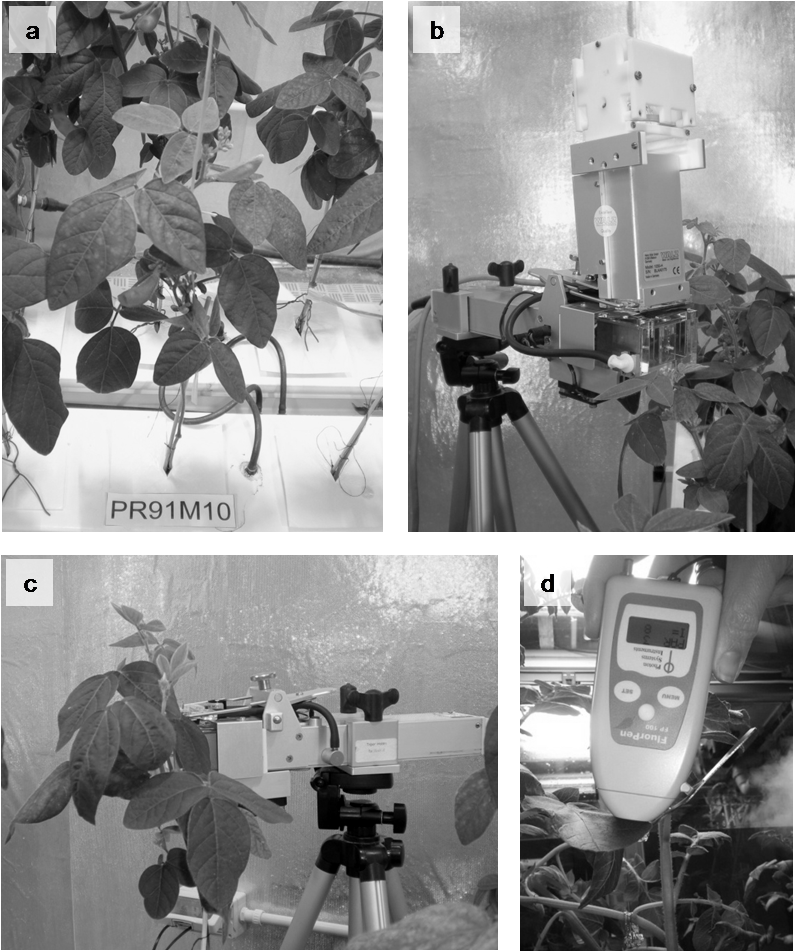

Supplement: FIGURE S1 — Plants of soybean cv. ‘PR91M10’ grown in closed-loop NFT (A). Measurements of photosynthesis with the Infra Red Gas Analyzer WALZ HCM 1000: particular of the halogen lamp for light response curves (B), and measurement at the ambient light intensity (C). Measurements of chlorophyll a fluorescence with the FluorPen FP100 max fluorometer (D). [file Image_1.TIF]
